# Design and application of secondary operation and maintenance supervision system based on AR modeling and indoor positioning

**DOI:** 10.1371/journal.pone.0290419

**Published:** 2023-10-25

**Authors:** Meng Han, Xianfei Zhou, Jianlin Jiao, Jiabo Chen, Kai Xu

**Affiliations:** State Grid Beijing Electric Power Corporation, Beijing, China; Sri Eshwar College of Engineering, INDIA

## Abstract

In order to facilitate the observation in the process of secondary equipment operation and maintenance supervision and the detection and tracking of operation and maintenance personnel, a secondary operation and maintenance supervision system based on AR modeling and indoor positioning is designed. The whole system is divided into seven levels and a unified information base, in which the basic level contains all kinds of secondary equipment; AR modeling layer uses augmented reality technology to create models for each secondary equipment in the basic layer, and determines the equipment position information based on ranging positioning technology; The data acquisition layer collects all kinds of original management data based on the constructed secondary equipment model; The data analysis layer reads and analyzes the information of the data acquisition layer through the data bus; The process support layer provides task scheduling support for the integrated management application based on the data analysis results; The integrated application layer uniformly monitors the secondary equipment based on the task scheduling results; The presentation layer is responsible for the interface presentation of all operation and maintenance and security management information of the system, and the unified information base provides data support for the whole system. The experimental results show that the secondary equipment model in the designed system has high definition, can obtain more image details, can realize the 3D display and real-time interaction of the secondary equipment operation and maintenance supervision results, and accurately mark the target and track for the staff.

## 1. Introduction

With the development of the times, people’s quality of life is constantly improving, and all walks of life put forward higher requirements for power supply [1]. Compared with the traditional substation, the intelligent substation is more economical and safe, and the power consumption is more reliable. In the intelligent substation, multi-functional intelligent electronic equipments and advanced communication networks are applied, so that the corresponding equipment of the secondary system can be effectively integrated, and the secondary system is required to have a higher level of operation and maintenance management [2]. Nowadays, the important development trend of substation construction is intelligent substation. The operation efficiency of intelligent substation will be directly affected by the quality of operation, maintenance and management [3]. Therefore, it is necessary to accurately grasp the characteristics of intelligent substation to effectively improve the work efficiency and economic benefits of substation. In the operation and maintenance management of substation secondary system, acceptance management, defect management and switching operation are very important contents. Strengthening the operation and maintenance process of substation can better control risks, integrate and optimize resources, and promote the more stable operation of intelligent substation.

In order to ensure the stable and reliable operation of the secondary system of power equipment, the routine inspection of the secondary system equipment in the substation has become a key technical means to ensure the safe operation of the power grid [4]. Among them, the substation power secondary equipment, such as switch handle, line sequence, terminal strip, pressing plate, indicator light, etc., need to be inspected at least once a month. At present, the inspection method of "paper + manual verification" has the problems of fatigue, error prone, man hour consumption and low efficiency.

The secondary system of intelligent substation has undergone fundamental changes compared with traditional stations in terms of equipment integration, data transmission mode and information acquisition mode [5]. Its maintenance test is difficult and the requirements for safety risk control of reconstruction and expansion are high. At present, the professional operation and maintenance management staff of substation relay protection are under great pressure. The relay protection equipment account, equipment defects, equipment inspection, protection action and other information are manually entered into the system. Due to large workload, low efficiency, inaccurate data and deviation from the actual situation, it has basically failed to meet the operation and maintenance requirements of the rapid development of intelligent substation, even the time of filling in the information is easy to be delayed, resulting in that the management personnel cannot grasp the actual situation in time and accurately [6], which has a serious impact on the secondary operation and maintenance management of the substation.

Han and Jiang et al. introduced the concept of knowledge data fusion in the operation management of power system equipment [7], and made full use of multi-source heterogeneous data to locate vulnerable processes, problem causes and influencing factors. Taking 2084 gas-insulated switchgear as an example, it is proved that the model is helpful to strengthen the management and control measures for the quality problems of power equipment. However, this model can not realize the 3D display and real-time interaction of the monitoring results of the operation and maintenance of secondary equipment. Aiming at the operation detection of substation secondary equipment, Zheng, Sun et al. studied the substation insulator infrared image detection system based on the improved single-excitation multi-box detector [8]. The system combines multi-scale feature mapping to generate a new feature pyramid, and designs a new feature enhancement module in the shallow network of the model, which improves the feature extraction ability of substation insulator infrared image. However, the system is error-prone and time-consuming. Yang and Liu et al. applied the improved random forest method to the operation and maintenance monitoring status assessment of substation secondary equipment [9], resampling the original data set at the generator end of the monitoring data acquisition (SCADA) system through bootstrapping to generate a decision tree. After weighting decision trees of different classification capabilities, IRF model is established to complete the evaluation of secondary equipment state. However, the system has some problems such as low efficiency and inaccurate data.

This paper designed a secondary operation and maintenance supervision system based on AR modeling and indoor positioning. The SOA architecture technology was used to design the overall structure of the secondary operation and maintenance monitoring system, and the augmented reality modeling module layer, data acquisition layer and patrol inspection management module were designed. The moving object recognition algorithm based on background difference was used to identify the secondary equipment maintenance personnel in the surveillance video image. AI technologies such as AR, indoor positioning, behavior perception and video dialogue are applied to the maintenance, defect and emergency services of the master station system, and auxiliary disposal mechanisms such as equipment status AR access, operation and maintenance work guidance and mobile collaborative operation are constructed to improve the disposal efficiency of operation and maintenance personnel, eliminate the blind area of operation and maintenance supervision, and improve the safety and intelligent protection level of operation and maintenance management and control.

## 2. Materials and methods

### 2.1 Overall structure of secondary operation and maintenance supervision system

The overall structure of the secondary operation and maintenance supervision system is designed by using SOA architecture technology and designed according to the layered architecture design idea [10], which realizes the separation of secondary equipment data acquisition and analysis processing, data analysis and processing and comprehensive application, and comprehensive application and display, so as to complete the overall architecture of the system and enhance the flexibility and expansibility of the system. In the system design, the principle and technology based on Service-Oriented Architecture (SOA) are used to emphasize the independence and loose coupling of services, so as to realize the independent development, deployment and upgrade of components. By defining clear service interfaces and protocols, each service can be developed and deployed independently of each other, thus reducing the complexity and coupling degree of the system. Using the service registration and discovery mechanism, services in the system can be dynamically registered and discovered. Supports the grouping of multiple services into more complex business processes or application scenarios.

**Fig 1:**
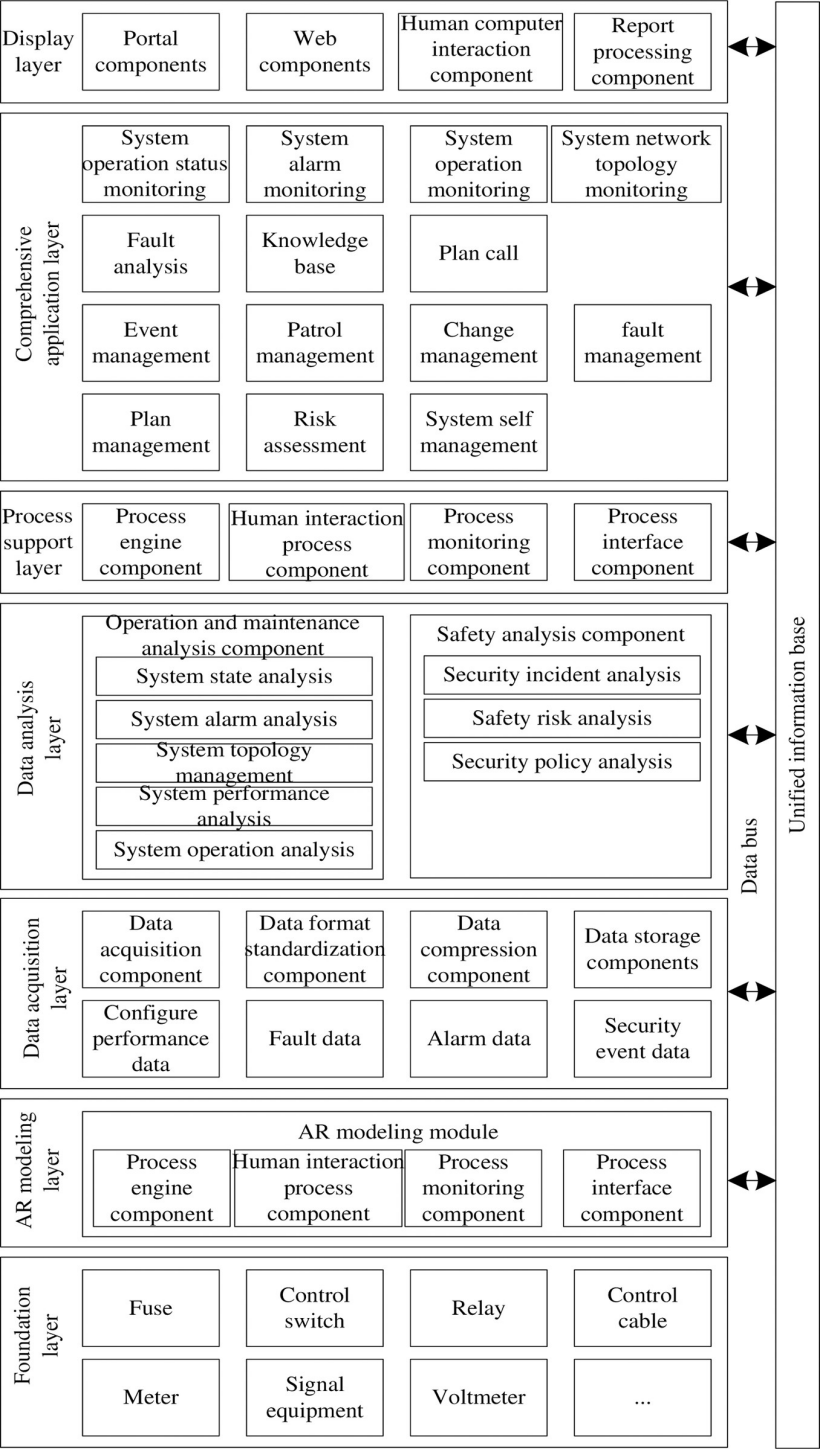
Overall structure of secondary operation and maintenance supervision system.

The overall architecture of the secondary operation and maintenance supervision system is divided into seven levels and a unified information base from bottom to top, namely: foundation layer, AR modeling layer, data acquisition layer, data analysis layer, process support layer, comprehensive application layer, presentation layer and unified information base. The overall architecture of the system is shown in Fig 1.

(1) The basic layer is the monitoring and management object of secondary operation and maintenance supervision and the main source of real-time data such as secondary equipment alarm. It mainly includes fuses, transformers, control switches, relays, control cables, instruments, signal equipment, automatic equipment, etc.

(2) The main function of AR modeling layer is to create models for each secondary equipment in the basic layer by using three-dimensional modeling technology. At the same time, it uses augmented reality technology and database technology to establish a rich standardized operation guidance library and operation inspection resource library. Determine the content and format of the standardized operation guide, design the database table structure to store the information of the standardized operation guide, determine the operation items and standards that need to be checked, design the database table structure to store the information of the operation check resource, create the database and set the appropriate access rights to ensure that only authorized users can access and modify the data, write the corresponding database query language (such as SQL) to create tables, insert, update and delete data, as well as query and retrieve information in the instruction library and resource library, identify the operation instruction library and operation check resources suitable for augmented reality technology, and use augmented reality technology to overlay or project the standardized operation instruction and operation check resources with the actual scenes of the physical world. It is applied to the operation and maintenance strategy of secondary equipment, and can effectively use the image recognition, intelligent error correction and process guidance technology of AR intelligent equipment, so as to improve the safety and efficiency of transportation inspection.

(3) The data acquisition layer is a data acquisition module located between the data analysis layer and the secondary equipment model. It follows the standard communication protocol to complete the collection of various original management data required by the system.

(4) The data analysis layer reads the performance, alarm, network, fault, safety event, configuration and other data contained in the data acquisition layer through the data bus. After aggregation and pre-defined configuration, the data bus distributes different data to different data analysis components for processing [11], which is to prevent the same data from being processed by different data processing component modules, resulting in repeated, redundant and different alarm and fault information.

(5) All process task scheduling of the system is based on a unified process engine. Based on the data analysis results, it provides the task scheduling support of management process such as event work order, problem management, change management and configuration management for the integrated management application. The Unified Process Engine is a tool for managing and executing business processes that provides functions such as modeling, scheduling, execution, and monitoring of business processes. Using business process modeling tools, such as BPMN or other similar standards, define and design the business processes that need to be executed. Configure the modeled business process into the unified process engine. Based on business requirements and time planning, the unified process engine is responsible for automatically triggering and executing related tasks and activities in a specified order or condition based on predetermined process definition and scheduling policies. Use the results of data analysis to schedule tasks.

(6) The integrated application layer realizes the unified monitoring of secondary equipment based on the task scheduling results, mainly including operation status monitoring, alarm monitoring, security event monitoring, system operation monitoring and network topology monitoring, so as to achieve the centralized monitoring of secondary equipment. When the operation and maintenance or decision-making layer finds the problems of secondary equipment through monitoring, it can use fault position analysis, knowledge base, plan call and database management to provide tool support functions to quickly and intelligently assist the operation and maintenance layer and decision-making layer to solve the problems of the system. When monitoring tasks in the integration application layer, the execution of tasks, state changes, and critical events can be recorded by inserting appropriate log statements into the application code. Using monitoring tools and frameworks, you can monitor the running status and key metrics of tasks in real time. For tasks involving network communication, network protocol analyzers and interface monitoring tools can be used to capture and analyze packets. For tasks that require transactional consistency, transaction monitoring techniques can be used to track the stages and states of transaction execution.

(7) The presentation layer is responsible for the interface presentation of all operation and maintenance and security management information of the system. It is the unified operation and maintenance presentation workbench of the comprehensive application layer and the interactive interface between the system and users. It mainly includes portal component, web page component, report processing component, human-computer interaction component, etc. The main goal of the presentation layer is to provide users with an intuitive, easy-to-use interface. It includes various UI components, such as buttons, text boxes, drop-down menus, tables, etc., for building the interface between the user and the system. Responsible for presenting data to users in a meaningful and understandable manner. The presentation layer is not just about presenting data, but also the ability for users to interact with the application. This includes user input validation, form submission, dialog box and alert handling, menu navigation, mouse and keyboard event handling, and more.

(8) The main characteristics of the unified information base are wide range of data, different requirements for real-time data, large amount of data, high security requirements and large amount of data processing. As the foundation and core of the system, it provides data support for the whole system and provides service functions such as data storage and maintenance, data synchronization, data archiving, object association query and so on.

### 2.2 Module layer design of AR modeling

**Fig 2:**
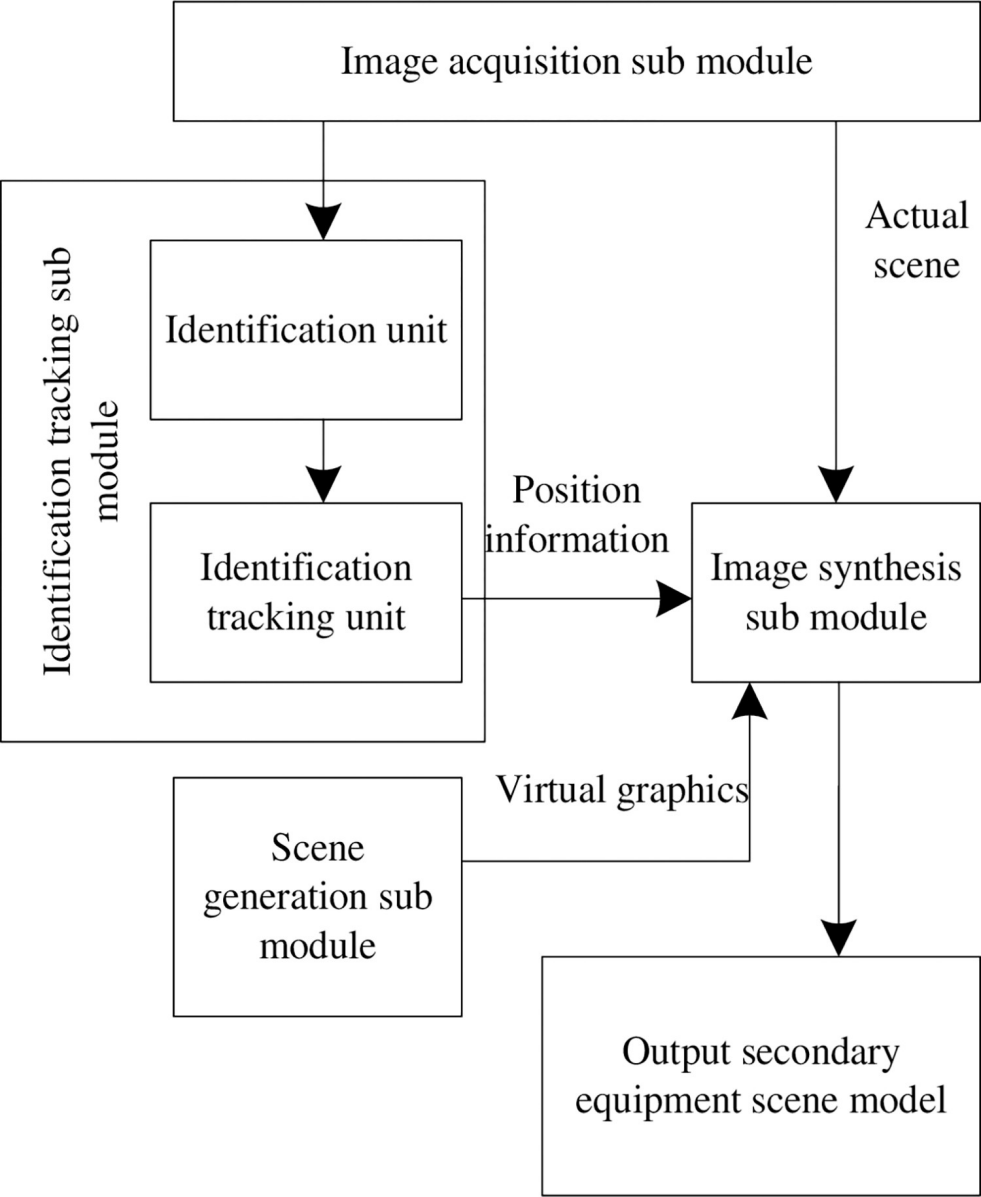
Framework structure of AR modeling module.

Augmented reality (AR) is a technology that calculates the position and angle of camera images in real time and adds corresponding images, videos and 3D models [12]. The goal of this technology is to enable the virtual world on the screen to combine and interact with the real scene. The AR modeling module consists of several hardware equipments and software. In order to simplify the design process of the module, the AR modeling module is divided into four sub modules: image acquisition sub module, identification and tracking sub module, scene generation sub module and display sub module. Fig 2 shows the framework of AR modeling module.

In the image acquisition sub module, the mobile equipment camera collects the video image of the secondary equipment, and the collected video image enters the identification recognition unit and image synthesis sub module in the identification recognition sub module respectively. After the identification recognition unit completes the identification of the secondary equipment, it indicates that the tracking unit uses the positioning technology based on ranging to track the identification of the secondary equipment, obtain the position information of the secondary equipment, and send the acquired position information of the secondary equipment into the image synthesis sub module; The scene generation unit generates the secondary equipment virtual model [13] according to the user interaction command. The generated equipment virtual model is also input into the image synthesis sub module, and is combined with the actual scene of the secondary equipment collected by the camera and the position information obtained by tracking in the sub module to output the secondary equipment scene model. The position information provided by the identification tracking unit for the image synthesis sub module is the basis for the seamless integration of the equipment virtual model in the mobile equipment camera video.

The identification tracking unit uses the ranging-based positioning technology to track the identification of the secondary equipment and obtain the position information of the secondary equipment. In this process, the positioning technology based on ranging [14] is mainly adopted, which determines the relative position of the secondary equipment according to the set relationship according to the distance between the unknown secondary equipment and the known secondary equipment. The core of this technology is ranging. The commonly used ranging methods are: received information strength indication method, signal arrival time difference method and signal round-trip time method.

(1) The received signal strength indication method is to determine the loss of the signal in the transmission process according to the signal strength received by the signal receiving secondary equipment based on the known transmitted signal strength of the signal transmitting secondary equipment [15]; Based on the signal propagation model, the secondary equipment positioning is carried out through the conversion between signal loss and distance. The logarithmic normal model of this method is:

$$L(d)=L(d_{0})+10n\log(\frac{d}{d_{0}})+Z_{\sigma }$$ (1)

In Eq (1), *L*(*d*) and *L*(*d*_0_) respectively represent the path loss away from the signal transmitting secondary equipment with distance *d* and the path loss after passing through the distance *d*_0_, and *n* and *Z*_*σ*_ respectively represent the path loss index and the noise with the mean value of 0. Based on Eq (1), the distance *d* is determined according to the path loss.

(2) The signal arrival time difference method is mainly that the signal transmitting secondary equipment transmits two wireless signals with different propagation speeds at the same time. The signal receiving secondary equipment can determine the distance *d* between the signal transmitting secondary equipment and the signal receiving secondary equipment according to the signal arrival time difference and the signal propagation speed. The time when the signal transmitting secondary equipment transmits two kinds of wireless signals is described by *T*_0_, the time when the signal receiving secondary equipment receives each signal is described by *T*_1_ and *T*_2_ respectively, and the propagation speed of the two signals is represented by *S*_1_ and *S*_2_ respectively, then the distance *d* can be described by the following equation:

$$d=T_{c}\times \frac{S_{1}\cdot S_{2}}{S_{1}-S_{2}}$$ (2)

In Eq (2), *T*_*c*_ represents the difference between *T*_2_ and *T*_1_.

The signal arrival time difference method has high ranging accuracy and can improve the problem of accurate time synchronization. It is relatively simple in the calculation process. When equipped with sensors, the system not only needs to increase the energy consumption, but also has the same cost of receiving signals.

(3) Using the signal round-trip time method for ranging is mainly based on the signal transmitting secondary equipment and signal receiving secondary equipment in different clock domains. The distance between the signal transmitting secondary equipment and the signal receiving secondary equipment is determined by determining the round-trip time and deducting the processing delay. The equation is described as follows:

$$\begin{array}{l} d=\frac{\left[\left(T_{3}-T_{2})+(T_{1}-T_{0}\right)\right]}{2}\times S\\ \;=\frac{\left[(T_{3}-T_{0})+T_{c}\right]}{2}\times S \end{array}$$ (3)

In Eq (3), *T*_0_, *T*_3_, *T*_1_ and *T*_2_ represent the clock domain of signal transmitting secondary equipment and signal receiving secondary equipment respectively.

In the actual positioning process of the system, selecting the ranging algorithm among the above three ranging algorithms can improve the measurement accuracy of the system. After determining the distance between the signal transmitting secondary equipment and the signal receiving secondary equipment, it can locate the secondary equipment through trilateral positioning and multilateral positioning algorithms.

The distance between the known equipment and the secondary equipment is calculated according to the secondary coordinates of the known equipment [16]. *A*(*x*_1_,*y*_2_), *B*(*x*_2_,*y*_2_), *C*(*x*_3_,*y*_3_) and *N*(*x*,*y*) are used to represent the coordinates of three reference secondary equipment and unknown secondary equipment respectively, and *j*_1_, *j*_2_ and *j*_3_ are used to represent the distance between three reference secondary equipment and unknown secondary equipment respectively, then the coordinates of unknown secondary equipment can be determined according to Eq (4):

$$\left\{ \begin{array}{l} {\left[\left(x-x_{1})+(y-y_{1}\right)\right]}^{2}=j_{1}{}^{2}\\ {\left[\left(x-x_{2})+(y-y_{2}\right)\right]}^{2}=j_{2}{}^{2}\\ {\left[\left(x-x_{3})+(y-y_{3}\right)\right]}^{2}=j_{3}{}^{2} \end{array}\right.$$ (4)

In Eq (4), the only intersection point of three circles can be determined by referring to the secondary equipment coordinate (*x*_*i*_,*y*_*i*_) and distance *j*_*i*_, that is, the unknown secondary equipment coordinate. The three circles in Eq (5) cannot intersect at the only point, so the maximum likelihood estimation method of multilateral positioning can be used to determine the coordinates of unknown secondary equipment. According to the multilateral positioning judgment *X* = *A*^−1^*b*, the coordinates of unknown secondary equipment can be determined as:

$$\left[\begin{array}{l} x\\ y \end{array}]={\left[\begin{array}{l} \left(x_{1}-x_{3})(y_{1}-y_{3}\right)\\ 2(x_{2}-x_{3})(y_{2}-y_{3}) \end{array}\right]}^{-1}\times [\begin{array}{l} x_{1}^{2}-x_{3}^{2}+y_{1}^{2}-y_{3}^{2}-j_{1}^{2}-j_{3}^{2}\\ x_{2}^{2}-x_{3}^{2}+y_{2}^{2}-y_{3}^{2}-j_{2}^{2}-j_{3}^{2} \end{array}\right]$$ (5)

The multilateral positioning algorithm based on trilateral positioning can make maximum use of the reference secondary equipment coordinates obtained by the unknown secondary equipment to determine its own position coordinates. (*x*_1_,*y*_1_), (*x*_2_,*y*_2_),… (*x*_*n*_,*y*_*n*_) are used to represent the position coordinates of the reference secondary equipment *A*_1_, *A*_2_,… *A*_*n*_, and *j*_1_, *j*_2_,… *j*_*n*_ are used to represent the distance from the unknown secondary equipment to different reference secondary equipment, then it can get:

$$\left\{ \begin{array}{c} {\left(x-x_{1}\right)}^{2}+{\left(y-y_{1}\right)}^{2}=j_{1}{}^{2}\\ {\left(x-x_{2}\right)}^{2}+{\left(y-y_{2}\right)}^{2}=j_{2}{}^{2}\\ \vdots \\ {\left(x-x_{n}\right)}^{2}+{\left(y-y_{n}\right)}^{2}=j_{n}{}^{2} \end{array}\right.$$ (6)

By optimizing Eq (6), Eq (7) can be obtained:

$$\left\{ \begin{array}{l} (x_{1}-x_{n})x+(y_{1}-y_{n})y=x_{1}^{2}-x_{n}^{2}+y_{1}^{2}-y_{n}^{2}-j_{1}^{2}-j_{n}^{2}\\ (x_{2}-x_{n})x+(y_{2}-y_{n})y=x_{2}^{2}-x_{n}^{2}+y_{2}^{2}-y_{n}^{2}-j_{2}^{2}-j_{n}^{2}\\ \;\vdots \\ (x_{n-1}-x_{n})x+(y_{n-1}-y_{n})y=x_{n-1}^{2}-x_{n}^{2}+y_{n-1}^{2}-y_{n}^{2}-j_{n-1}^{2}-j_{n}^{2} \end{array}\right.$$ (7)

Eq (7) is converted into the thread equation form *AX* = *b*, and the unknown secondary equipment coordinate (*x*,*y*) can be determined based on MMSE estimator *X* = (*A*^*T*^*A*)^−1^*A*^*T*^*b* of *X*. Through the above process, the position information of secondary equipment can be accurately determined.

### 2.3 Design of data acquisition layer

**Fig 3:**
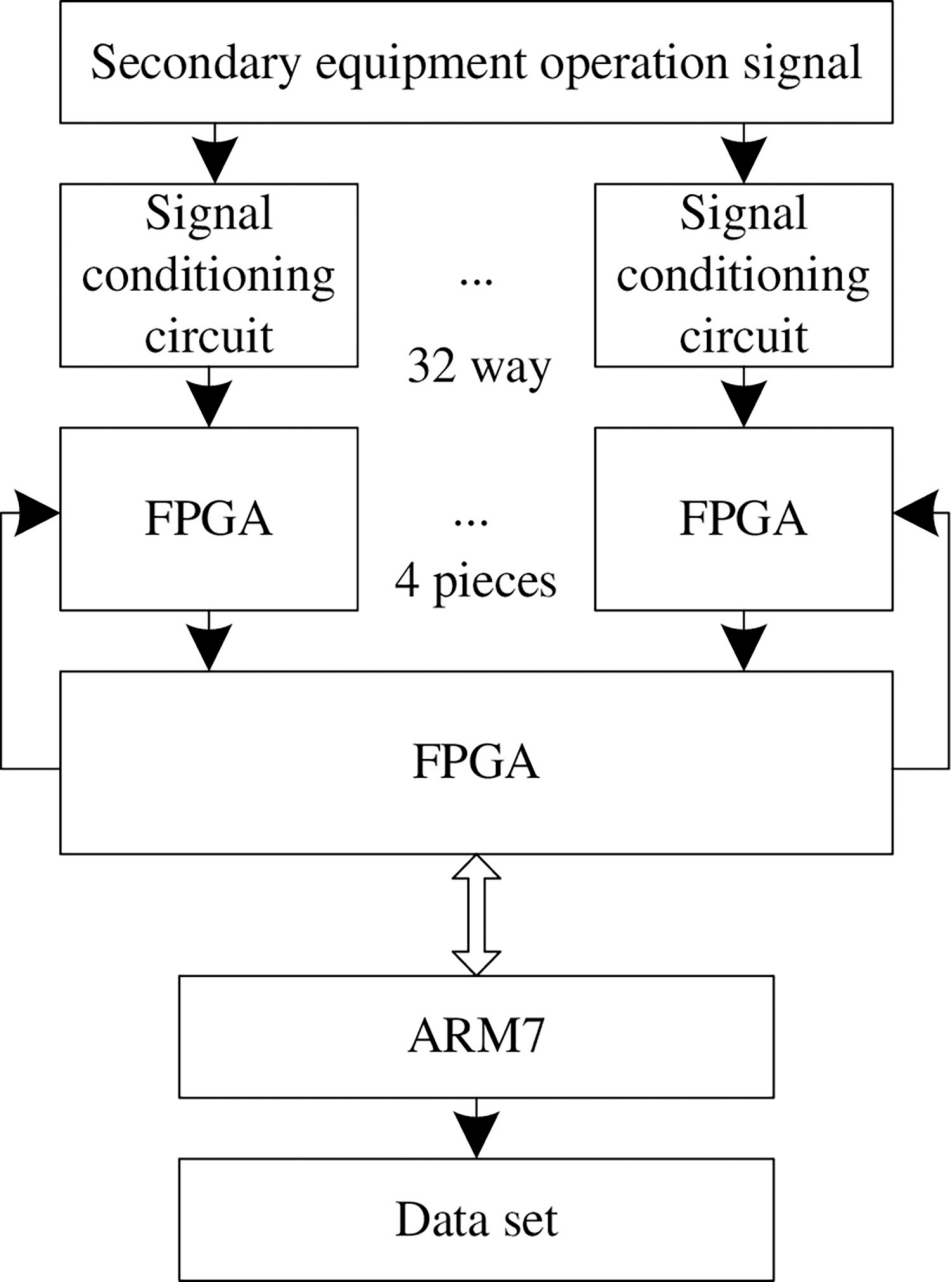
Structure diagram of data acquisition layer.

According to the design requirements, the overall structure of the data acquisition layer is determined, as shown in Fig 3. The data acquisition layer is mainly composed of signal conditioning circuit, ADC and its peripheral circuit, FPGA and its peripheral circuit, ARM and its peripheral circuit.

After the operation signal of the secondary equipment enters the ADC (analog-to-digital converter) through the signal conditioning circuit, the analog signal of the secondary equipment is converted into digital signal, and the digital signal is transmitted to FPGA (field programmable gate array) for storage; The ARM processor reads the operation information of the secondary equipment stored in the FPGA. Under the control of the embedded control software, the analysis algorithm in the chip is used to classify the operation information of the secondary equipment and store it in different data sets.

ADC chip is one of the core equipment of data acquisition layer, which completes the conversion from analog signal to digital signal of secondary equipment [17]. Considering the requirements for the signal range, sampling accuracy, sampling rate and number of channels of the tested secondary equipment, AD9222-50 of AD company is selected as the ADC chip. This chip is an 8-way, 12 bit, 50MSPS analog-to-digital converter (ADC), which meets the design requirements.

Considering the requirements of I / O quantity, logic unit, storage resources and working clock in this design, EP3C80F484C8 chip in Cyclone III series of Altera company is selected for FPGA. Because the acquisition module does not need to cache a large amount of sampling data, the requirements of sampling depth can be met by using the internal storage resources of FPGA.

S3C4510B of SamSung company is selected as ARM processor, which is a low-cost ARM7 chip specially designed for network applications. At present, the embedded module based on the chip has been successfully developed. The module expands all control signals of S3C4510B processor through two high-density MOLEX-53481G connectors [18], which is suitable for rapid construction of the system with ARM7 processor as the core.

The embedded control software selects uClinux operating system. Compared with VxWorks and other systems, its biggest advantage is that the code is all open source, which is especially suitable for running on microcontrollers without memory management unit (MMU). In order to adapt to embedded applications, uClinux has compact code, optimized performance, simple and flexible functions and high utilization of storage resources, which makes uClinux small, stable performance, supporting a variety of file systems, powerful network functions and good portability.

### 2.4 Design of patrol inspection management module

**Fig 4:**
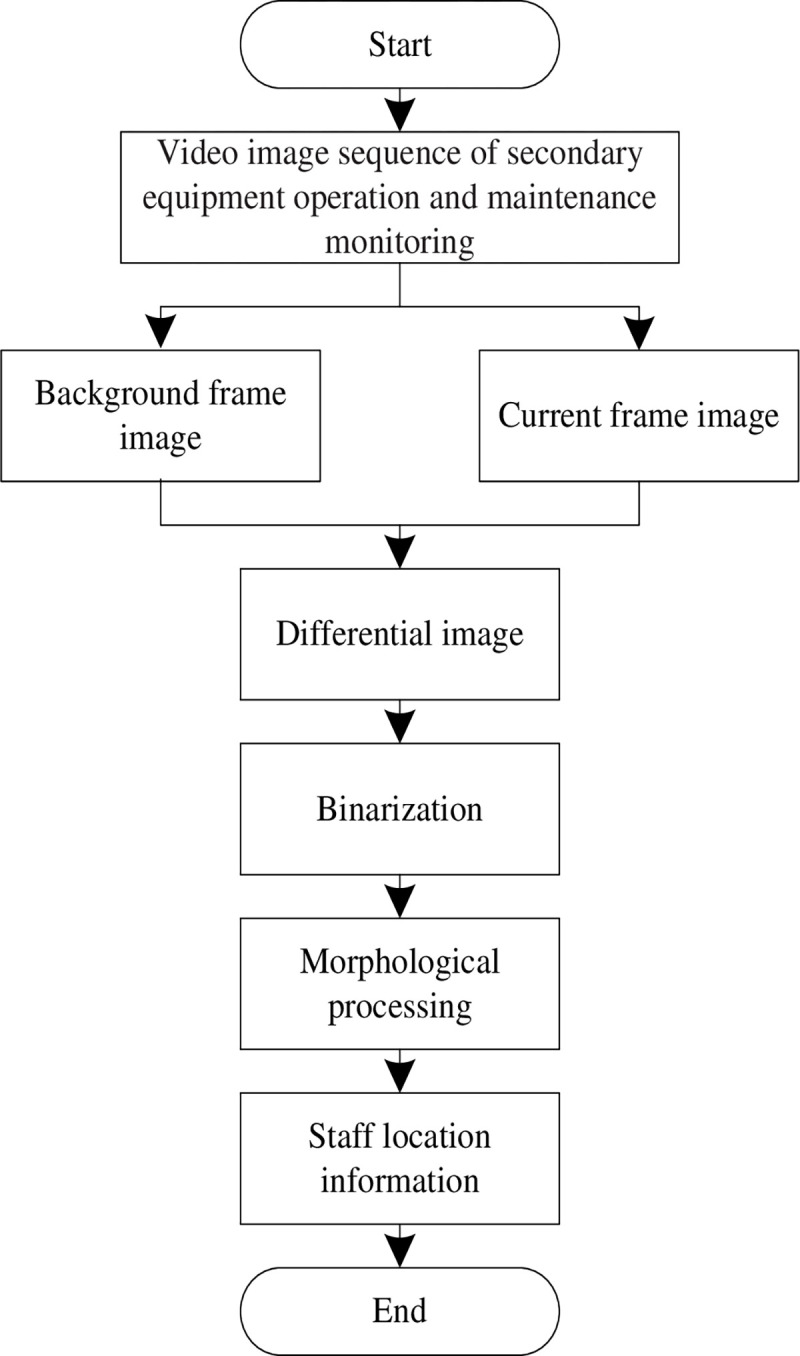
Flow chart of moving target recognition algorithm based on background difference.

In the patrol inspection management module in the comprehensive application layer, the moving target recognition algorithm based on background difference is used to identify the secondary equipment maintenance personnel in the monitoring video image [19]. In the inspection management module, the moving target recognition algorithm can provide basic data for AR applications. By identifying and tracking moving targets, the relevant information is superimposed in real time in the real scene for users to observe and operate. This can provide more intuitive, real-time inspection information to help users better understand and deal with the scene situation. Therefore, the combination of moving target recognition algorithm and AR technology can improve the interaction and visualization effect of the inspection management module, and further improve the efficiency and accuracy of the inspection work. Fig 4 shows the flow of moving target recognition algorithm based on background difference.

(1) The background image of secondary equipment operation and maintenance monitoring video is initialized;

In the process of background initialization, firstly, the background of secondary equipment operation and maintenance monitoring video image is modeled. *G*_*t*_ is set as the observation value at time *t* for a certain pixel (*i*,*j*) in the video image sequence of secondary equipment operation and maintenance monitoring. For a series of observation values {*G*_1_, *G*_2_,⋯,*G*_*t*_} at a given point (*i*,*j*), it can be regarded as a random statistical process independent of other points. *B* Gaussian distribution mixture models are used to simulate, then the probability distribution of (*i*,*j*) at time *t* is:

$$P(G_{t})=(\sum_{b=1}^{B}w_{b,t}\times N(G_{t},\sum_{b,t}))$$ (8)

$$N(G_{t},\Sigma_{t})=\frac{e^{-\frac{1}{2}{\left(G_{t}\right)}^{T}\sum_{t}^{-1}\left(G_{t}\right)}}{{\left(2\pi \right)}^{\frac{n}{2}}{\left|\Sigma_{t}\right|}^{\frac{1}{2}}}$$ (9)

(2) The algorithm in this paper is used to extract the changed part in the video sequence image of secondary equipment operation and maintenance monitoring, and binarize it;

The above processes are used to read the pixels of the secondary equipment operation and maintenance monitoring video image, update and extract the background. On this basis, the background is differentially processed to extract the outline of secondary equipment maintenance personnel [20–22]. The current frame image is represented by *f*_*b*_(*x*,*y*) and the background frame is *f*_*bb*_(*x*,*y*) [23, 24], so the differential image can be represented by Eq (11).


$$D_{b}(x,y)=[f_{b}(x,y)-f_{bb}(x,y)]\times \phi$$ (10)

In Eq (10), *ϕ* represents the correction coefficient [25, 26].

The binary image is represented by Eq (11):

$$R_{b}(x,y)=\{\begin{array}{l} 1,D_{b}(x,y)\geq T\\ 0,D_{b}(x,y)<T \end{array}$$ (11)

The obtained difference image is binarized according to Eq (11). When a pixel in the difference image is greater than the threshold [27, 28], it is considered that the pixel is the foreground, otherwise, it is the background.

(3) Morphological processing is carried out on the above processed image to remove small noise points [29], and fill the holes and connection breakpoints inside the moving target at the same time, so as to obtain a complete moving area and extract the moving target.

## 3. Results

In order to verify the practical application effect of the secondary operation and maintenance supervision system based on AR modeling and indoor positioning designed in this paper on the secondary operation and maintenance supervision, the secondary equipment in a substation is taken as the test object, to build this system, and this system is used to supervise the operation and maintenance of the test object. The test results are as follows.

### 3.1 Operation and maintenance supervision results

**Fig 5:**
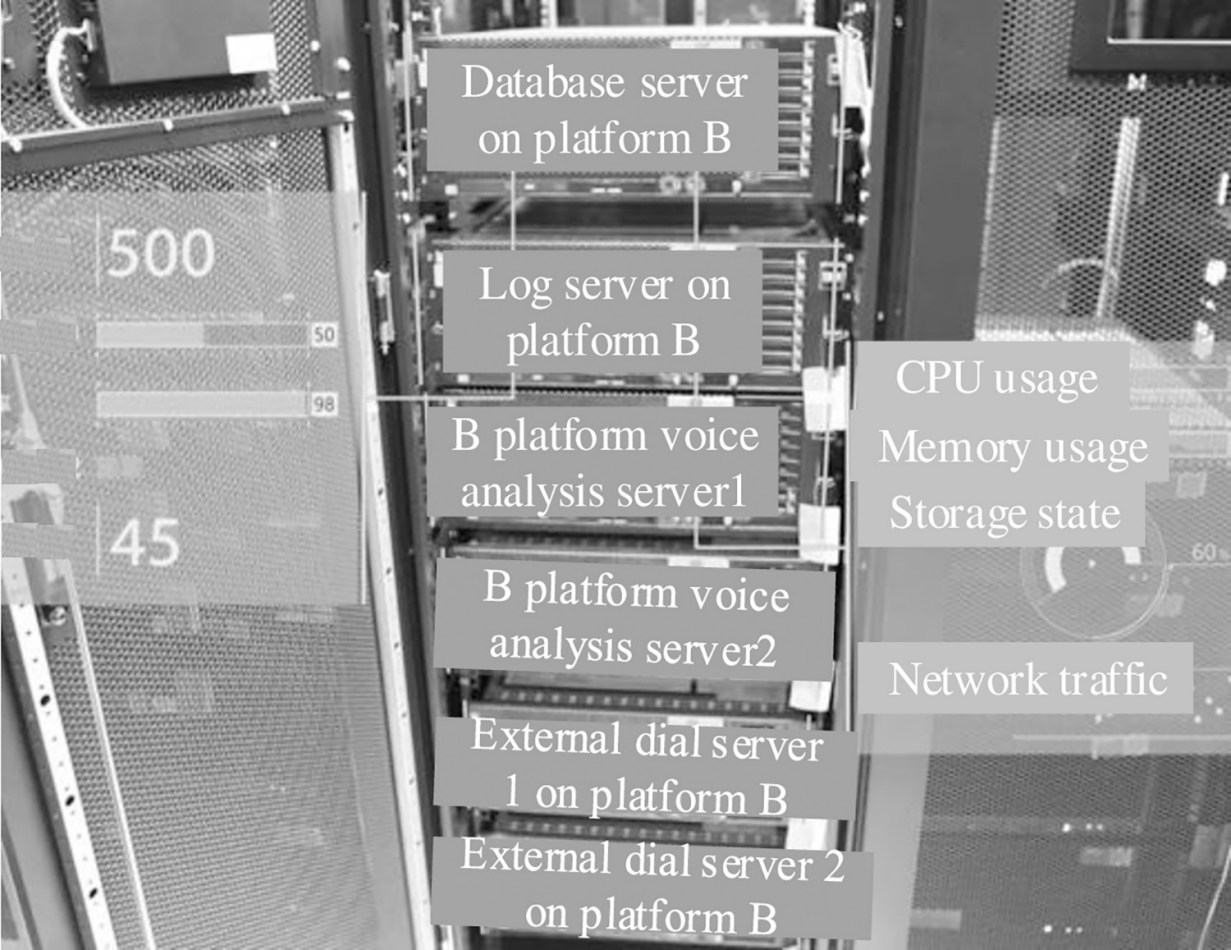
Schematic diagram of AR patrol inspection.

Fig 5 shows the presentation of the operation and maintenance supervision results of the system for the test object.

According to the analysis of Fig 5, the system in this paper can effectively realize the 3D display and real-time interaction of the operation and maintenance supervision results of the experimental object. The augmented reality inspection platform includes the database server on platform B, log server on platform B, voice analysis server on platform B, voice analysis server on platform B, external dial-up server on platform B, external dial-up server on platform B, CPU usage, memory usage, storage status, and network traffic. In this paper, the system expands the display screen to the real environment, so that the computer window and icon are superimposed on the real object, and the three-dimensional object can be presented in the user’s panoramic view according to the current task or needs. At the same time, its shape and appearance can be adjusted as needed, and the virtual scene can be superimposed on the real scene. In this paper, the main station side of the system can configure the equipment information that the AR tool needs to receive according to the business needs. After the configuration is successful, the information acquisition center will automatically synchronize the collected equipment signals, operation parameters and alarms to the AR terminal, so as to facilitate the end user to observe the target equipment. The realization process of AR modeling: using LiDAR, structured light scanner or camera and other equipment to collect data on the real scene. The collected data is transformed into a point cloud, and the collected point cloud is registered and aligned to make it fuse into an overall three-dimensional model. Based on the registered point cloud data, a 3D reconstruction algorithm is used to generate a geometric model. Add material and texture information to geometric models to make them more realistic. Texture images can be taken with a camera, or external images can be combined with the model using a mapping algorithm. The generated 3D model is refined and optimized. Deploy the resulting model to an AR device, such as AR glasses. Through sensors, tracking algorithms and graphics rendering technology, 3D models are overlaid with real-time images or videos to achieve augmented reality effects.

### 3.2 Modeling results

#### 3.2.1 Model presentation

**Fig 6:**
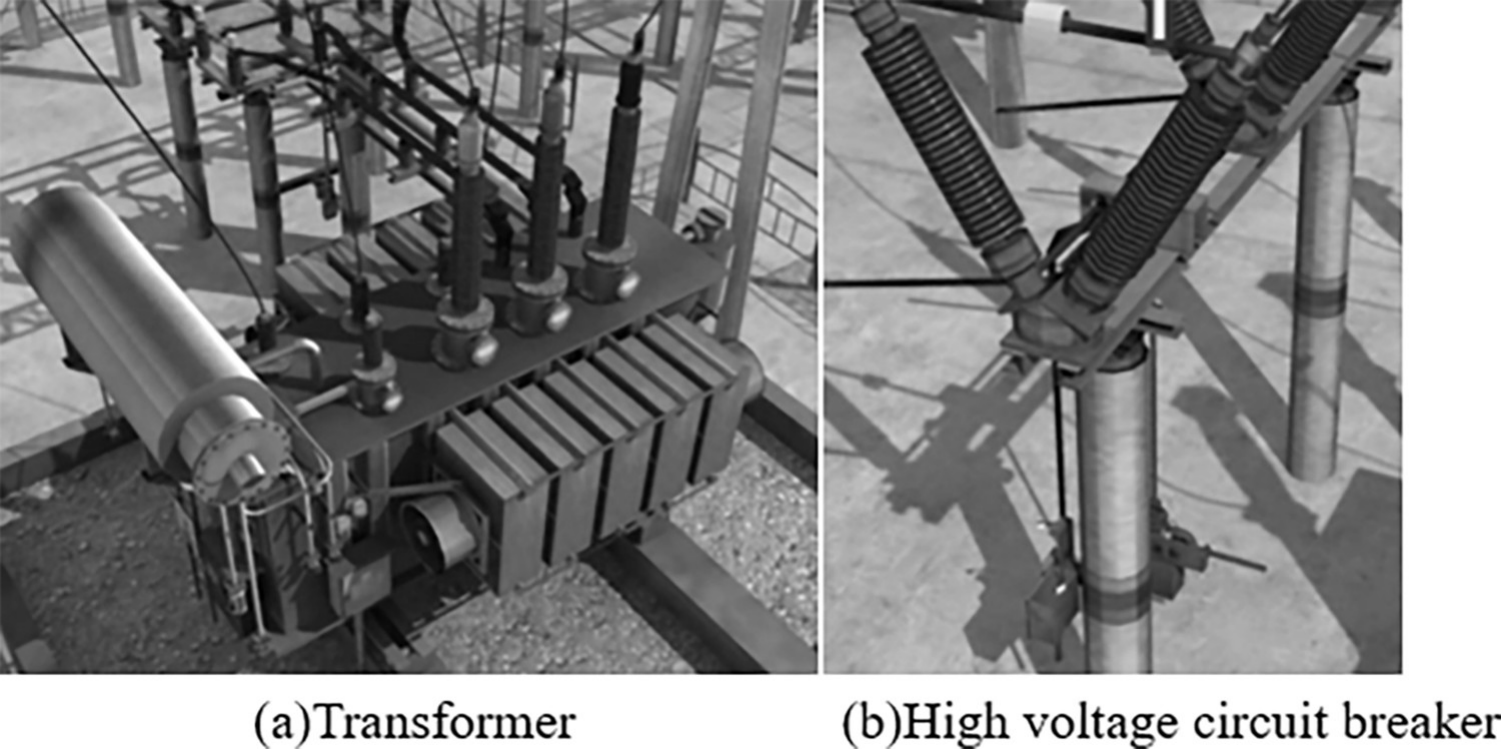
AR modeling effect. (a)Transformer (b)High voltage circuit breaker.

The AR modeling of the test object is carried out by using the method in this paper. The model construction results of transformer and high-voltage circuit breaker in the test object are shown in Fig 6.

According to the analysis of Fig 6, the AR modeling of the test object using the method in this paper can more truly present the actual situation of the test object, with high definition and can obtain more image details, which shows that the method in this paper is applicable.

#### 3.2.2. Comparison of modeling effect

**Fig 7:**
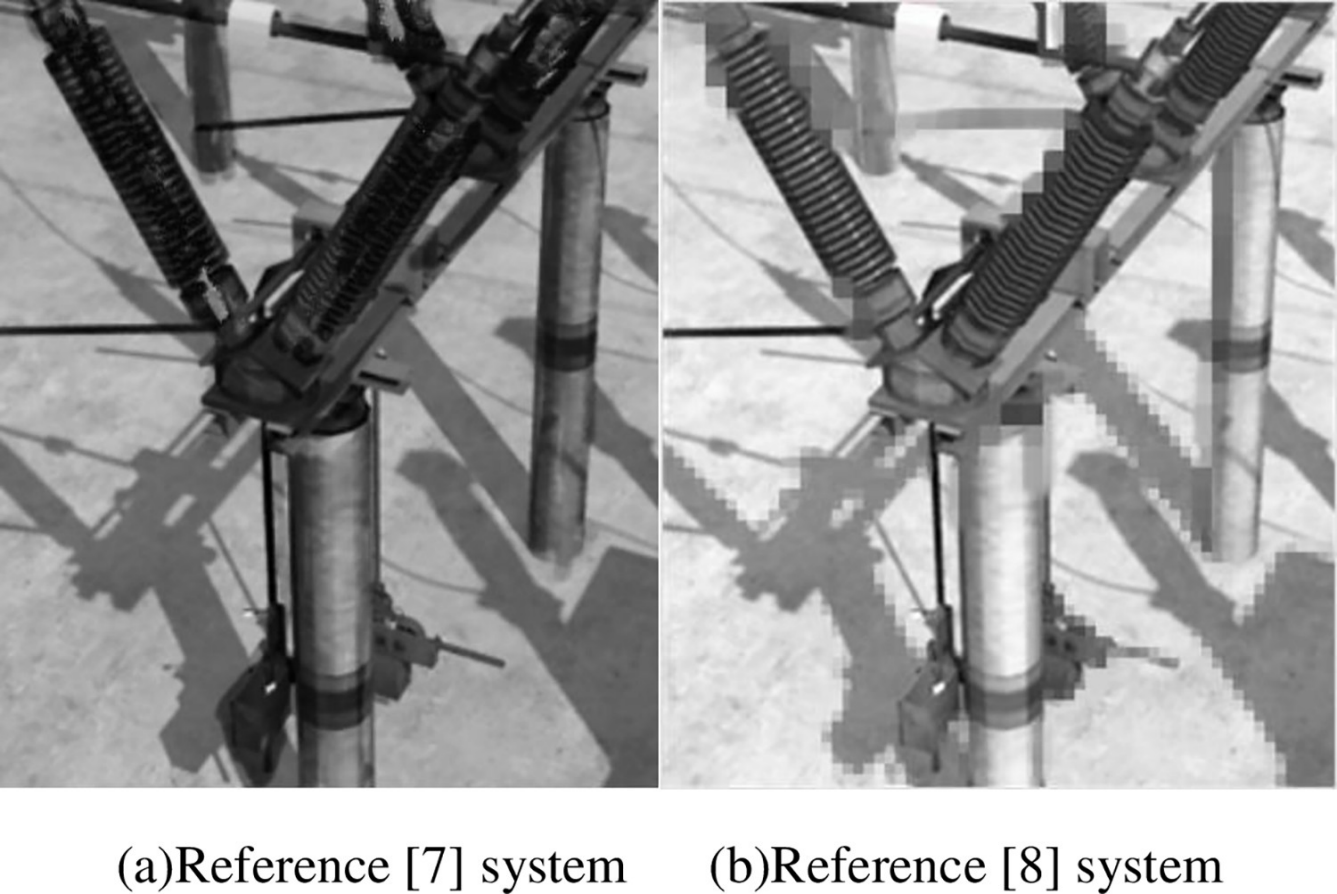
Comparison of system modeling results. (a)Reference [7] system (b)Reference [8] system.

In order to further illustrate and verify the modeling effect of the method in this paper, the AR model of high-voltage circuit breaker shown in Fig 6(B) is taken as the standard, the knowledge-based data fusion system in reference [7] and the system based on improved multi box detector fused single excitation in reference [8] are used as the comparison systems, to construct the three-dimensional model of the same target. The results are shown in Fig 7.

It can be seen from the modeling results of the three systems in Figs 6(A) and 7, compared with the system in this paper, the system modeling results in reference [7] have poor structural treatment for dark areas, some areas have deep colors, some details are weakened, and even some details are lost. There is a serious image virtualization problem in the system modeling results in reference [8]. Although the brightness has been improved to a certain extent, the overall exposure of the three-dimensional model is high and the overall effect is not clear. Therefore, the modeling effect of the system in this paper is better than the two comparison methods.

### 3.3 Personnel identification results

**Fig 8:**
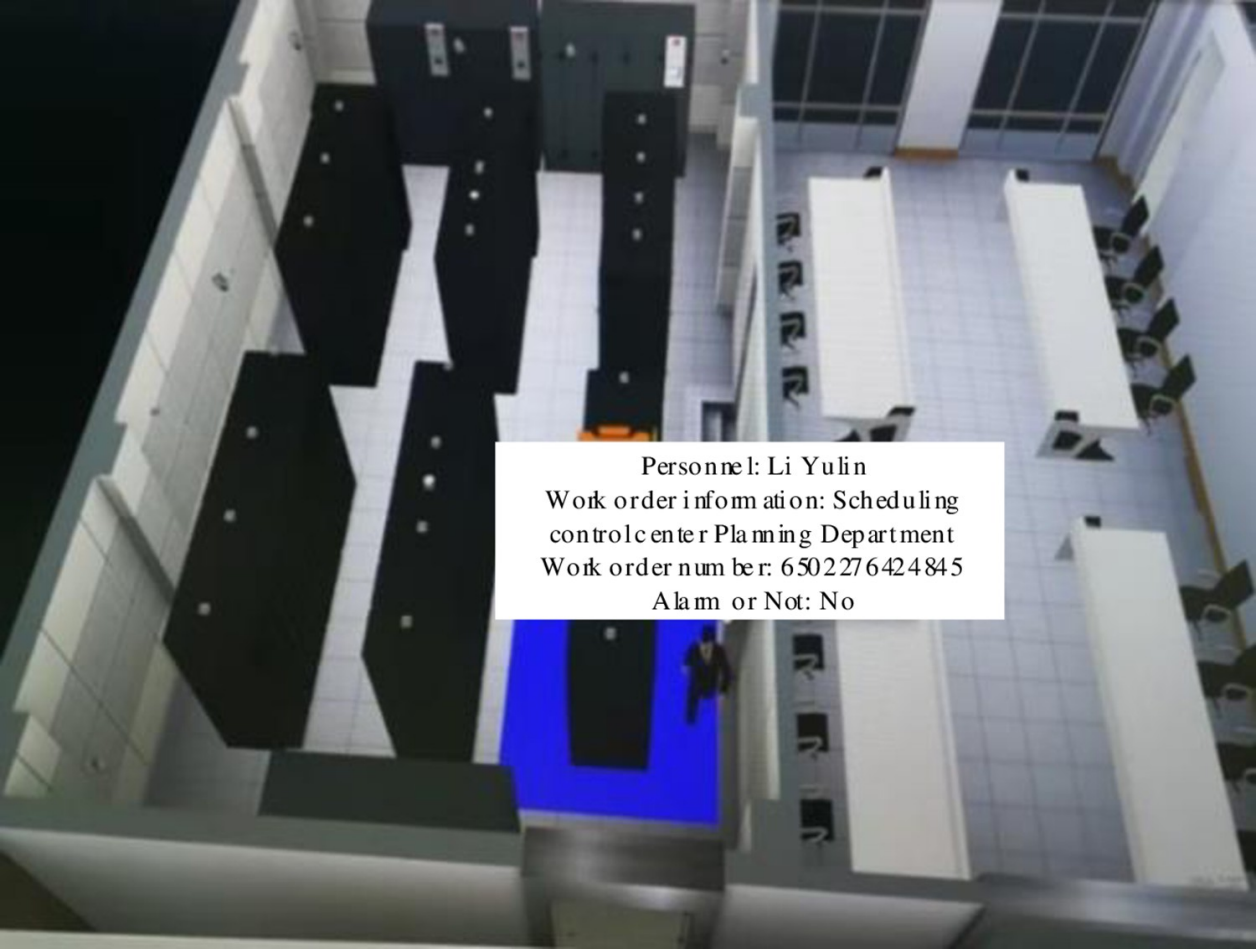
Staff information identification results.

Fig 8 shows the position information recognition results of the system for staff in this paper.

According to the analysis of Fig 8, through the integrated intelligent video analysis, the system in this paper identifies, detects and analyzes the video image of the operation and maintenance of the test object, filters out the interference, and marks the target and track of the staff in the video image of the operation and maintenance monitoring of the secondary equipment. For the track rationality analysis of operation and maintenance personnel, the original manual verification mode is changed to the mode of automatic real-time verification and timely alarm of the system, so as to improve the efficiency, accuracy and timeliness of supervision. At the same time, the system in this paper can detect and track the operation and maintenance personnel. Each camera can monitor a variety of different targets at the same time, and can set a virtual fence (electronic fence) to standardize the activity range of personnel in different roles.

### 3.4 Analysis of average outage probability

**Fig 9:**
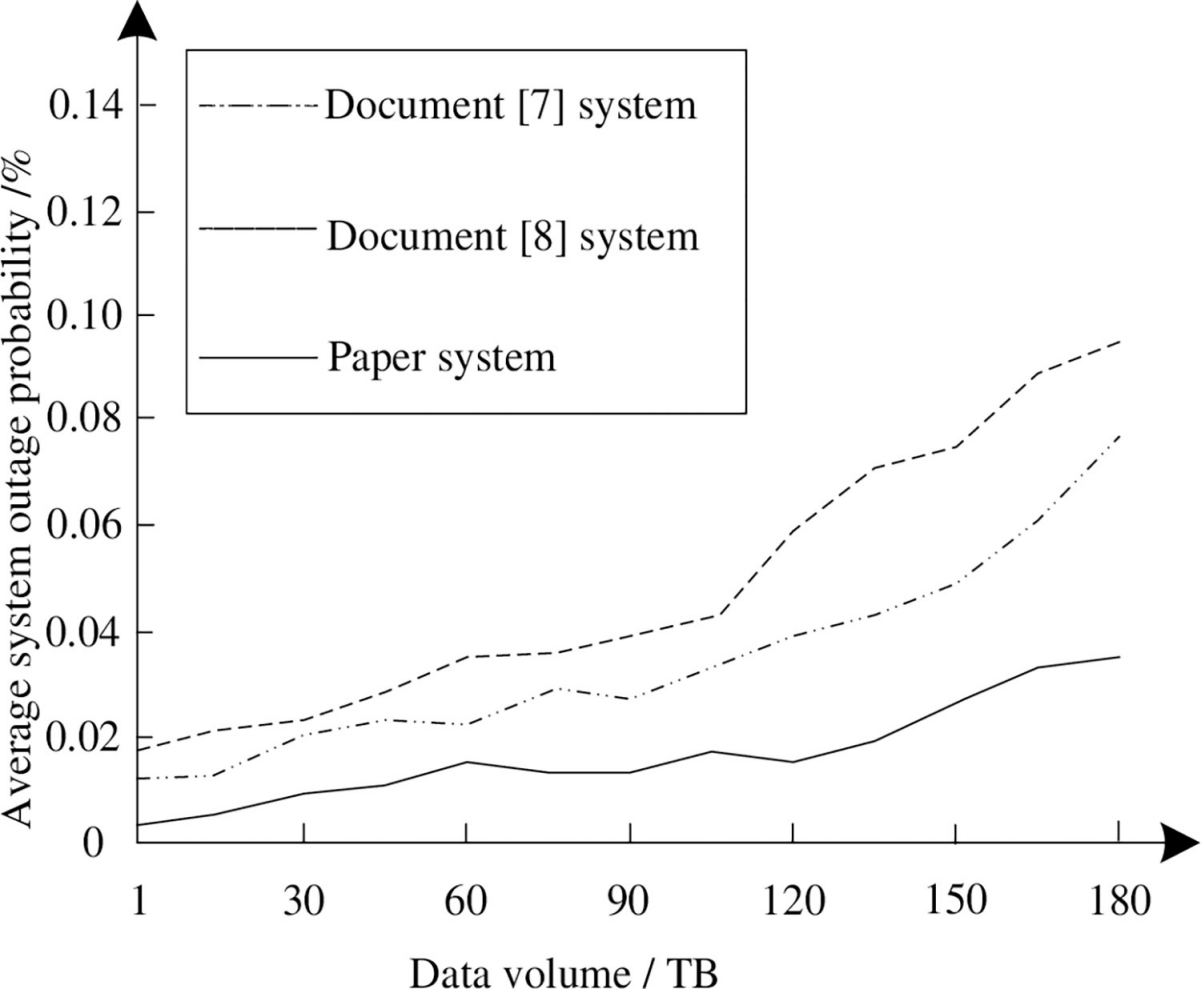
Average outage probability of different systems.

Average outage probability is one of the important indexes to characterize the reliability of distribution system. This indicator can reflect the severity and importance of power distribution system outage. In order to verify the stability of the system in this paper, the interruption probability of the system in this paper, the system in reference [7] and the system in reference [8] under different data volume conditions is recorded in the same experimental environment. The results are described in Fig 9.

By analyzing Fig 9, it can be seen that when the amount of data does not exceed 60 TB, the average outage probability of the system in this paper is not much different from that of the other two systems; When the amount of data exceeds 60 TB, the average outage probability of the three systems increases with the increase of the amount of data, when the data volume is 180 TB, the average interrupt probability of the system in this paper is 0.038%, and that of the other two systems is 0.098% and 0.076%. But compared with the other two systems, the average outage probability curve of the system in this paper increases and changes more gently, indicating that the system in this paper has better stability.

### 3.5 Fault diagnosis test

Ten secondary equipment are randomly selected in the experimental object, and the system in this paper is used for fault diagnosis of their operation state. Table 1 shows the fault diagnosis results of the selected secondary equipment.

**Table 1:** Fault diagnosis results.

Secondary equipment	Actual fault	This paper presents the system diagnosis results
Fuse	Short circuit fault	Short circuit fault
No. 1 control switch	Contact burning	Contact burning
Transformer 1	Winding fault	Winding fault
Relay 1	Contact looseness and cracking	Contact looseness and cracking
Transformer 2	Iron core silicon steel sheet short circuit	Iron core silicon steel sheet short circuit
No. 2 control switch	Normal state	Normal state
Control cable	Connector box water inlet	Connector box water inlet
Relay 2	Glass insulator damage	Glass insulator damage
Signal equipment	Normal state	Normal state
Relay 3	Normal state	Normal state

The analysis of Table 1 shows that the fault state detection results of each secondary equipment in the experimental object using the system of this paper are completely consistent with their actual faults, which shows that this system has strong application performance.

### 3.6 Indoor positioning accuracy

**Fig 10:**
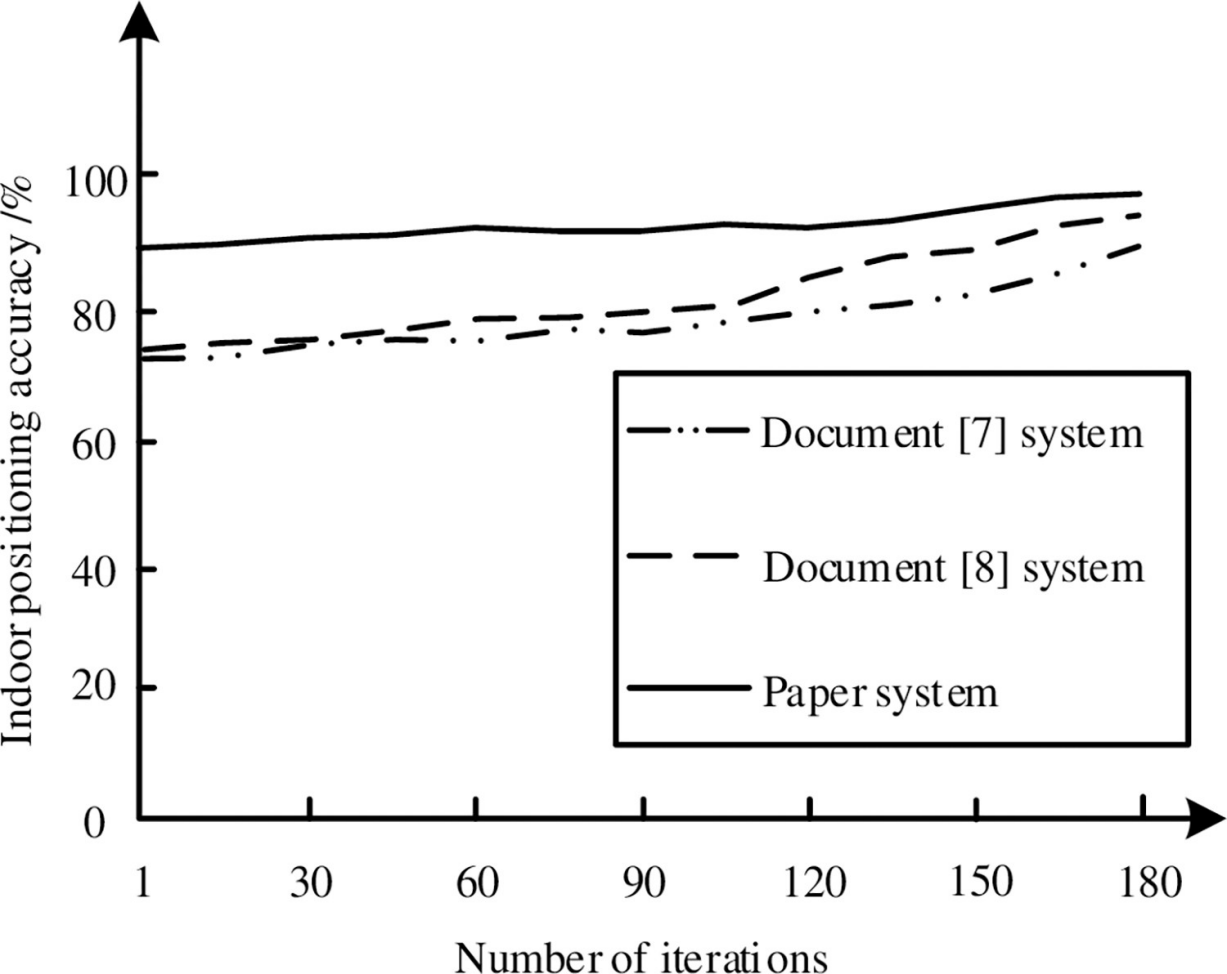
Indoor positioning accuracy of different systems.

The accuracy of indoor positioning is usually affected by a number of factors, including the accuracy of the sensor, the accuracy of the map data, the algorithm of data synchronization and processing. In order to further verify the effectiveness of the system in this paper, the system in this paper is compared with the system in literature [7] and the system in literature [8], and the specific comparison results are shown in Fig 10.

As can be seen from Fig 10, the indoor positioning accuracy of the system in this paper is the highest 95%, while the indoor positioning accuracy of the two literature systems is lower than 90%. Compared with the two literature systems, the indoor positioning accuracy of the system in this paper is higher.

## 4. Conclusion

This paper designs the secondary operation and maintenance supervision system based on AR modeling and indoor positioning, applies AR technology and indoor positioning technology to the operation and maintenance supervision of substation secondary equipment, and verifies the application performance of the system designed in this paper through experiments. The AR modeling layer uses augmented reality technology to model each secondary device in the base layer, and determines the location information of the device according to the range-locating technology. The data acquisition layer collects all kinds of original management data according to the constructed secondary device model. The data analysis layer reads and analyzes the information of the data acquisition layer through the data bus; The integrated application layer monitors the secondary devices in a unified manner according to the task scheduling results. The presentation layer is responsible for the interface representation of all operation and security management information of the system, and the unified information base provides data support for the entire system. The results show that the system can achieve the expected design purpose. However, due to the limitation of time and technology, there are still the following deficiencies in the field application of the system designed in this paper:

1. The intelligent terminal is not integrated into the helmet, which is not conducive to operation and maintenance.
2. The correlation between QR code data of background system and equipment is not high.
3. The data source is single, the sample data is insufficient, and the effect of on-site auxiliary function is not obvious.

In view of the above problems, the comprehensive promotion and application of the system also needs to further improve the functions of hardware terminal equipment, continuous data accumulation, fusion defect automatic diagnosis and so on, so as to better improve the operation efficiency. Some potential limitations and obstacles may arise during the implementation of a proposed system: scalability challenges when the system needs to handle large numbers of users, large amounts of data, or high frequency of requests. The system may not be able to efficiently scale and handle loads beyond its capacity range. When integrating a new system into an existing infrastructure, you may face compatibility and interface issues with existing systems. The introduction of a new system may face user acceptance challenges. Strategies to mitigate these challenges include the adoption of appropriate architectures and technologies, such as distributed systems, cloud computing, load balancing, etc., to provide high availability and resilient scalability. When implementing system integration, adequate planning and testing is carried out to ensure compatibility with existing infrastructure. Standardized interfaces and data formats are adopted, and the integration process between systems is simplified through API and protocol adaptation. The implementation of the system is phased to reduce risk and minimize impact on existing business. Through iterative improvement and user feedback, the system functions and user experience are gradually improved.
